# Uncovering the structure of self-regulation: a network analysis among German primary-school children

**DOI:** 10.3389/fpsyg.2026.1823651

**Published:** 2026-07-20

**Authors:** Petra Warschburger, Robert Busching, Christian Gericke, Birgit Elsner, Annette Maria Klein, Rebecca Bondü

**Affiliations:** 1 Department of Psychology, University of Potsdam, Potsdam, Germany; 2 International Psychoanalytic University Berlin, Berlin, Germany; 3 Psychologische Hochschule Berlin, Berlin, Germany

**Keywords:** age, binary sex, childhood development, network analysis, self-regulation facets, socioeconomic status

## Abstract

**Introduction:**

Self-regulation plays a pivotal role in development, contributing to overall health and success. Self-regulation encompasses several facets, both basal and complex in nature, whose interaction is not fully understood.

**Methods:**

The current study investigated the network structure of a wide range of self-regulation facets (i.e., cognitive, behavioral,, physiological; basal and complex; performance-based or affective questionnaires) in 1,657 German primary-school children at 6–11 years and again three years later.

**Results:**

At both measurement points, network analyses revealed a similar structure of two main clusters of basal self-regulation facets (one affective, one cognitive), which were linked by the complex facet planning. At neither measurement point did the network structure differ by binary sex or age (above/below 8.4 or 11.1 years, respectively), but it differed by parental education at the first measurement point. Physiological self-regulation (heart rate variability), as well as the complex facets delay of gratification and affective decision-making showed no connections to the other self-regulation facets.

**Conclusion:**

The results add to research on childhood development by indicating differentiation of the self-regulation facets across three years, with the complex facet planning playing a central role within the self-regulation network, evident by close links to clusters of affective and cognitive basal self-regulation facets. Furthermore, our data suggest that changes in structures need sufficient time to unfold in middle childhood. Although the (lacking) effects of binary sex, age, and parental education require further investigation, our results offer key insights into the self-regulation network and in childhood development, which could inform future interventions.

## 1 Introduction

Self-regulation is the general ability to control and manage one’s physiology, emotions, cognition, and behavior to achieve important personal aims (Baumeister and Vohs, 2004; Nigg, 2017; de Ridder et al., 2017) and is usually considered to encompass emotional, cognitive and behavioral regulation (Nigg, 2017). As such, self-regulation is an umbrella term that encompasses a broad range of competencies, such as heart rate variability (HRV), anger regulation, inhibition, cognitive flexibility (flexibility), or delay of gratification. There is an ongoing debate about the definition and structure of self-regulation - including executive functions - in childhood and adolescence (Bridgett et al., 2013; Zhou et al., 2012). It has been suggested that self-regulation facets can be categorized into basal and complex facets (Warschburger et al., 2023). In this paper, we define basal self-regulation facets as physiological (e.g., HRV), cognitive [executive functions; i.e., inhibition, flexibility, working-memory updating (updating)], and affective (e.g., emotional reactivity) competencies that enable basic psychological functioning and likely show hereditary influences (Freis et al., 2022). In addition, we assume that the orchestration of these basal facets enables more complex self-regulation, which includes the abilities to delay gratification or to make rational decisions when faced with tempting cues, or to plan behavior in order to reach longer-term goals and shield this behavior from affective impulses. Regarding development, it is assumed that self-regulation facets continue to differentiate, but also integrate, as they mature (e.g., Bailey and Jones, 2019; Ibbotson, 2023; Wesarg-Menzel et al., 2023). The present study examined whether the theoretically derived internal structure of basal versus complex, as well as of physiological, affective, behavioral, and cognitive self-regulation facets can be empirically identified in middle childhood. Via network analyses, we aimed to identify core self-regulation facets and important interrelations, to examine influences of binary sex, age, and parental education; and to investigate changes in the identified self-regulation network structure across three years from middle childhood to early adolescence.

### 1.1 The network structure of self-regulation

To date, empirical research identifying the structure of self-regulation and its sub-facets is limited. Two of the most important challenges are the lack of a common definition of self-regulation and the broad range of self-regulation measures (Nigg, 2017). Consistent with most of the proposed conceptual and taxonomic classifications, this study assumes a hierarchical structure in which executive functions - as basal, cognitive competencies - provide a basis for more complex self-regulation skills, such as planning (Bailey and Jones, 2019; Nigg, 2017; Warschburger et al., 2023). So far, most research has focused on single self-regulation facets or small subsets thereof, particularly in samples of child and adolescent participants with still developing skills (Robson et al., 2020). In addition, different self-regulation facets with the same name can capture different contents, and self-regulation facets with substantial content overlap are sometimes given distinct names (e.g., Duckworth and Kern, 2011; Enkavi et al., 2019). To identify the structure of self-regulation competencies, many studies have relied on zero-order correlations (see Karr et al., 2018), which often fail to hold once additional variables are included in the model. Factor analytic models as a more appropriate approach have yielded important, but oftentimes inconsistent results (e.g., regarding the structure of executive functions; Karr et al., 2018), and have been criticized for assuming a latent causal construct underlying the structure of self-regulation facets, or the independence of self-regulation facets (Willoughby et al., 2014). In contrast, network analyses use partial correlations to visualize relation patterns, providing a more comprehensive understanding of complex psychological phenomena like self-regulation (Eisenberg et al., 2018; Karr et al., 2022; Rosales et al., 2023). Network analyses are data-driven and exploratory, and they account for the complexity and partly overlapping nature of self-regulation facets. The present study is the first to apply this approach to the structure of self-regulation during childhood and its assumed developmental changes.

One of the key questions in self-regulation research is whether self-regulation is a coherent psychological construct that is reflected by the diverse self-regulation facets and different assessment approaches. In their seminal work, Eisenberg et al. (2019) used 37 performance-based tasks and 23 self-report questionnaires, combining different methodological approaches (e.g., exploratory factor analysis; network/similarity-based clustering) to analyze the structure of self-regulation in a sample of 522 adults (aged 18–63 years). In their study, no unified self-regulation factor emerged – a pattern that was replicated also in other studies (Holmén et al., 2024; Neubeck et al., 2022). Instead, Eisenberg et al. (2019) identified two unrelated modality-based clusters of self-regulation competencies using network analysis. The clusters primarily reflected different methodological approaches - one cluster included questionnaire-based and the other cluster performance-based measures. Of note, the performance-based measures corresponded to our definition of basal self-regulation facets, while the questionnaire-based measures aligned with complex self-regulation facets. Moreover, the cluster of questionnaire data modestly predicted real-world outcomes, but the other cluster did not. This suggests that the two-cluster structure is not just a methodological artifact of the included measurement methods but rather reflects differences in the underlying self-regulation capacities that are assessed with behavioral tasks or questionnaires, respectively. In line with Eisenberg et al. (2019), Neubeck et al. (2022) also did not find a single latent self-regulation trait using network analysis. Instead, their results indicated a differentiation between facets of self-regulation [primarily reflecting complex self-regulation; see (Warschburger et al., 2023)], measured with well-established questionnaires (e.g., emotion regulation, impulsiveness, self-control), and basal, cognitive executive functions, measured with performance-based tasks (e.g., updating, inhibition, shifting/flexibility). This aligns with the observation of Eisenberg et al. (2019) that questionnaires and performance-based (mainly executive functions) assessments do not measure a single latent self-regulation trait. Of note, by estimating separate networks for different age groups (*N* = 333; 14–24, 25–49, >50 years) they also reported developmental changes in the organization of self-regulation networks from loosely connected systems in the younger age group to a more integrated regulatory network with more interconnections between the “executive-functions” and the “self-regulation cluster” in the middle-aged group. In contrast to these findings, Holmén et al. (2024), focusing only on performance-based tasks measuring inhibition, flexibility, and updating, did not find support for a meaningful latent organization of these different executive functions. While Neubeck et al. (2022) observed that self-control variables connected the “executive functions” (performance-based) and the “self-regulation cluster” (esp. questionnaire-based emotion regulation) and self-control emerged as highly central, Holmén et al. (2024) found no central node. By combining different statistical approaches, these studies underline the importance of network analyses when testing theoretical assumptions about the internal structure of basal and complex self-regulation facets assessed with different measures. In addition, Neubeck et al. (2022) provided preliminary insight into the age-related variability of self-regulation networks, although children, a particularly relevant age group for self-regulation development, were not considered.

While Eisenberg et al. (2019) and Neubeck et al. (2022) have taken a comprehensive approach by including basal and complex self-regulation facets, network analyses among children have almost exclusively focused on executive functions as basal, cognitive facets of self-regulation, mostly inhibition, flexibility, and updating. Research suggests that throughout development, these core executive functions (Miyake et al., 2000) form an increasingly interactive system that promotes more complex self-regulation (Bailey and Jones, 2019; Karr et al., 2022; Warschburger et al., 2023). Overall, the literature converges on a developmental model suggesting that self-regulation networks can best be viewed as a re-organization of the interconnections among several self-regulation facets: progressing from weakly differentiated systems in early childhood into a more efficient, hierarchically organized system with increasing functional specialization in later childhood and adolescence. Hartung et al. (2020) consistently showed, by using three different statistical approaches, -moderated factor analysis, structural equation modeling, and network analysis-that executive functions (working memory, working memory updating, flexibility, inhibition) become increasingly differentiated between ages 7 and 15. This general pattern of increasing separation and differentiation was also replicated in further network analyses (Karr et al., 2022; Menu et al., 2024; Rosales et al., 2023). Younger et al. (2023) indicated that this development was characterized less by the emergence of new relevant components and more by the refinement and strengthening of existing network organizations. Additionally, Menu et al. (2022) showed that cognitive training can accelerate this process, with younger children (aged 9–10) benefiting more than adolescents (aged 15–17). This suggests the existence of sensitive periods for the development of self-regulation competencies. In line with that conclusion, several studies (Rosales et al., 2023; Younger et al., 2023) reported greater fluctuations in children’s networks compared to those of adolescents.

A major question arises as to which facets of self-regulation can be drivers in this re-organization process. Centrality measures in network analysis provide crucial insights into the structures and dynamics of a network, and can be instrumental in processes of change. These measures identify key actors who, by virtue of their network positions, have the potential to exert influence. So far, several studies have analyzed centrality measures, yielding differing results. While Hartung et al. (2020) concluded that increasing age-related differentiation was driven by growing independence of inhibition, a finding that underscores its central role particularly in younger age groups, Menu et al. (2024) reported a developmental transition from inhibition-centered networks (children) to updating-centered networks (adolescents). Conversely, Karr et al. (2022) found that from ages 3–10, all three executive functions were equally central, whereas in early adolescence, flexibility emerged as the most central variable and as a mediator between inhibition and working memory updating. Notably, the only network analysis in this specific context that focused exclusively on adults, conducted by Holmén et al. (2024), strongly contrasts with the aforementioned network research in children and adolescents suggesting a meaningful structure of connectivity. Contrary to these studies, they found no clear indication of a central variable among inhibition, working memory, and flexibility, and only weak and unstructured relations between the different performance-based tasks. However, Neubeck et al.’s (2022) network analysis extended this approach by integrating cognitive and affective domains within a single network framework. Their findings suggest that self-control acts as a central bridging node linking cognitive executive functions and emotion regulation processes. Importantly, this work emphasizes increasing integration across development, suggesting that regulatory systems become more coordinated rather than strictly segregated. In conclusion, previous studies suggest that self-regulation, and specifically its cognitive domain involving executive functions, should not be conceptualized as a single unified construct but rather as a complex system with multiple levels, whose structures vary across different developmental stages and measurement modalities.

To summarize, there is limited and contradictory evidence regarding the network structure of self-regulation, including executive functions as basal, cognitive facets, particularly with respect to the numbers and contents of the identified communities and central variables that connect different clusters. Moreover, different measures for the same self-regulation or executive-function facet do not necessarily show high inter-correlations or belong to the same cluster. Our knowledge of age-related differences is mainly based on cross-sectional comparisons between broader developmental periods. Research on children and adolescents has primarily focused on the three “core” executive functions, suggesting a differentiation (i.e., less close relations between different executive function constructs) with increasing age, and highlighting the proposed functional separation and specialization of basal self-regulation facets during development (Miyake et al., 2000). However, there is a lack of data on the multidimensionality and complexity of self-regulation (Dias et al., 2024; Larraín-Valenzuela et al., 2022; Nigg, 2017), on factors influencing self-regulation network structures, and on age-related differences across childhood. As self-regulation develops and can be applied to more complex demands and situations, different facets emerge and differentiate along distinct developmental trajectories (e.g., Fernández-García et al., 2023; Vink et al., 2020; Wesarg-Menzel et al., 2023). Therefore, it is important to examine interrelations of a variety of different self-regulation facets as well as age-related changes of the identified networks in self-regulation development.

### 1.2 The present study

We aimed to address these research gaps by identifying the network structure of 10 self-regulation facets as well as developmental changes in the networks across three years from childhood to adolescence. In particular, we chose self-regulation facets that had been identified as being important in previous developmental research (e.g., Robson et al., 2020), and we extended that research by including seven basal facets (including executive functions like inhibition, flexibility, updating) and three complex facets (planning, affective decision making, delay of gratification). We included not only measures of cognitive and behavioral, but also of physiological and affective self-regulation, and we combined data from parent/teacher questionnaires and performance-based tasks. With network analyses, we aimed to analyze complex patterns and the varying strength of relationships between the facets, to identify core features within the network, and recognize the complexity and interdependence of the self-regulation facets. We used data from two measurement points three years apart, derived from a large population-based sample of 1,657 children in middle childhood (aged 6–11 years at T1; aged 9–13 at T3). Importantly, our data enabled the examination of the stability and dynamics of the internal structure of basal and complex self-regulation-facets across 3 years from middle childhood into adolescence.

Table 1 provides an overview of how we categorized the ten examined self-regulation facets according to complexity (i.e., basal, complex), primary focus (e.g., affective, cognitive), and source of data collection. Although network models are exploratory and data-driven, we formulated three research questions (RQ) and anticipated several relations based on prior research.

**TABLE 1:** Overview of included self-regulation (SR) facets.

SR facet	Complexity	Domain	Source	Measurement point	
				T1	T3
Emotional reactivity	Basal	Affective	Parent-report	✔	✔
Anger reactivity	Basal	Affective	Parent-report	✔	n.a.
Inhibition	Basal	Cognitive	Performance-based	✔	✔
Inhibitory control	Basal	Cognitive (behavioral)	Parent-report	✔	✔
Updating	Basal	Cognitive	Performance-based	✔	✔
Flexibility	Basal	Cognitive	Performance-based	✔	✔
Heart rate variability	Basal	Physiological	Objective measurement	✔	n.a.
Delay of gratification	Complex	Affective, cognitive, behavioral	Performance-based	✔	✔
Affective decision-making	Complex	Affective, cognitive, behavioral	Performance-based	✔	✔
Planning	Complex	Cognitive, behavioral	Teacher-report	✔	✔

n.a., not assessed at T3.

### 1.3 RQ1: Which network structure for the investigated self-regulation facets can be found in middle childhood (T1; age 6–11 years)?

We expected to find multiple clusters that are connected with each other either directly or indirectly via specific facets. The exact composition of these clusters is difficult to predict, because the variables (i.e., the nodes) representing the self-regulation facets could cluster by basal vs. complex facets, by affective vs. cognitive vs. physiological vs. behavioral foci, or by questionnaires vs. performance-based tests.

### 1.4 RQ2: Is this network structure of self-regulation facets moderated by binary sex, age group, or socioeconomic status (SES)?

There is abundant evidence that self-regulation competencies differ as a function of age (e.g., Wesarg-Menzel et al., 2023), that they vary by binary sex (with mixed results regarding a female or male advantage for various facets, e.g., Gaillard et al., 2021; Grissom and Reyes, 2019), and are negatively related to socioeconomic status (SES) or parental education in particular (e.g., Fernández-García et al., 2021; Wesarg-Menzel et al., 2023). However, much less is known about whether these sociodemographic variables also impact the internal structure of self-regulation facets. With respect to age, we expected a higher degree of differentiation of the network structure (i.e., weaker correlations between the self-regulation facets) at older compared to younger age. We did not find any network analyses that examined effects of sex or SES/parental education. Therefore, the present study addressed an important research gap by investigating potential differences in the identified self-regulation network structures when splitting our sample by binary sex, median age, or lower/higher parental education (as a proxy for SES).

### 1.5 RQ3: Does the network structure of self-regulation facets differ across 3 years?

Besides the well-known increase in the mean levels of different self-regulation facets (e.g., Wesarg-Menzel et al., 2023), several studies have shown age-related changes in the network structure of self-regulation or executive functions (e.g., Karr et al., 2022; Menu et al., 2024), pointing to an increasing differentiation of (cognitive) self-regulation competencies throughout childhood and adolescence. Therefore, the internal structures identified by the network analysis at the two measurement points should also differ. In particular, we expected a further differentiation of the executive functions, as basal self-regulation facets, indicated by a decreased number and strength of relations (i.e., edges) between the facets. We also expected a stronger role of complex self-regulation facets with increasing age.

## 2 Materials and methods

### 2.1 Sample

We included data from two measurement points of the PIER study (“Potsdam Intrapersonal Risk Study”; Warschburger et al., 2023). T1 (first measurement) was conducted in 2012–2013 and included data of 1,657 children (52% girls, *M*_*age*_ = 8.36, SD = 0.95; min = 6.2, max = 11.32; grades 1–3), T3^1^ (about 3 years later) included data of 1,534 children (52% girls, *M_*age*_* = 11.01, SD = 0.92; min = 9.12; max = 13.75; grades 4-6). Data were collected in 33 primary schools with 120 classes in the German federal state of Brandenburg, covering rural and urban areas with different social stratification. Approximately 65% of the children had at least one parent with higher education (e.g., a secondary school diploma equivalent to the German *Abitur*). The order of the assessments was block-randomized and assessments were conducted in two individual sessions. A logistic regression with the participation at T3 as dependent variable and all mentioned T1 variables as predictors revealed no systematic differences between the samples at T1 and T3. The only exception was binary sex (with a small effect of 51.6% girls at T1 and 52.1 % girls at T3), indicating that girls were slightly more likely to participate at T3.

### 2.2 Measures

#### 2.2.1 Sociodemographic information

Sociodemographic data at both measurement points included the assessment of sex (female, male), age of the child, and of SES with parental education as a proxy. For the moderation analyses, we used binary sex, dichotomized age at the median (T1: ≤ vs. >8.4 years; T3: ≤ vs. >11.1 years), and split parental education between higher (e.g., secondary education certificate; German: Abitur) vs. lower school degrees.

#### 2.2.2 Self-regulation facets

We assessed 10 self-regulation facets via multiple measurement tools, aiming to capture the multidimensional nature of self-regulation (see Table 1). The employed instruments are described in further detail in our study protocol (Warschburger et al., 2023). With the exception of anger reactivity and HRV (both T1 only), all data were available at both T1 and T3.

##### 2.2.2.1 Basal self-regulation facets

*Emotional reactivity, anger reactivity*: We assessed these two sub-facets to measure emotional reactivity. At T1 and T3, parents answered 10 items of the Behavior Rating Inventory of Executive Function (BRIEF; Gioia et al., 2000) emotional-control subscale, ranging from (1) *never* to (5) *always*. At T1, parents answered 7 items of the Temperament in Middle Childhood Questionnaire (TMCQ; Simonds, 2006) anger/frustration subscale, ranging from (1) *totally disagree* to (5) *totally agree*. We calculated a mean score for each sub-facet, general emotional reactivity and anger reactivity, with higher scores indicating lower self-regulation.

*Inhibition, inhibitory control*: We measured performance-based inhibition with the Fruit Stroop (paper-based; Archibald and Kerns, 1999). In each of four trials, participants had to name the true color of the presented 25 items as quickly as possible according to varying rules, and we measured time (seconds). We computed the interference score time Trial4−([time Trial1 × time Trial3]/[time Trial1 + time Trial3]) (Archibald and Kerns, 1999), inverted on their mean so that higher values indicate higher self-regulation. Additionally, parents reported on 6 items of the TMCQ (Temperament in Middle Childhood Questionnaire, Simonds, 2006) inhibitory-control subscale to measure children’s ability to suppress primary behavioral impulses, ranging from (1) *true* to (5) *false.* We computed a mean score, with higher scores indicating higher self-regulation.

*Updating*: We measured updating with the Digit Span Backward task from the German version of the HAWIK-IV test of intelligence (Petermann and Petermann, 2010) to capture children’s ability to recall and modulate given information. Children were to repeat verbally presented digit spans of increasing length in reverse order. Starting with only two digits, children had to answer at least one of the spans per length correctly in order to proceed to a 1-digit longer span (maximum length: 9 digits). We computed a sum score of all correct trials, with higher scores indicating higher self-regulation.

*Flexibility*: At T1, we used the computer-based Cognitive Attention Shifting Task (Röthlisberger et al., 2010). In 46 trials with inter-stimulus intervals of 300–700 ms, children saw one single- and one multi-colored fish on each screen side. Children had to alternatingly feed the single- and the multi-colored fish by pressing predetermined letters on a QWERTZ-keyboard. As the sides changed in 22 switch trials, children had to remember their previous response and change their response pattern accordingly. We computed the sum of the correct responses in all trials, with higher scores indicating higher self-regulation. At T3, we used the computer-based Dimensional Change Card Sorting task (Qu et al., 2013). Presented with two geometrical symbols varying in shape and color (e.g., red star, yellow square), children had to pair a third symbol (e.g., yellow star) to one symbol according to a verbally announced rule (“shape” or “color”) as fast as possible via keypress. In the first 20 trials, a dominant rule was established (“shape” or “color” for half of the participants, respectively). Then, 45 non-switch trials following the dominant rule and 15 switch trials following the non-dominant rule appeared pseudo-randomly. We computed switch-costs from accuracy, with lower scores indicating higher self-regulation.

*Heart-rate variability (HRV)*: We recorded resting state HRV at T1 with Polar Watches (Model RS800CX). For 3 minutes, children placed their wrists on the electrodes, which were lightly taped to the belt to avoid movement-induced measurement interruptions. We instructed children to breathe normally and move as little as possible. We computed HRV as the root mean square of successive time difference between two heart beats (RMSSD; Friedman et al., 2002) using the software provided by Polar together with the watches. We included only cases with plausible values (RMSSD < 160, heart rate between 55 and 120, and a low-to-high frequency < 4). Higher scores indicated higher self-regulation.

##### 2.2.2.2 Complex self-regulation facets

*Delay of gratification*: We used four decision tasks to measure children’s ability to delay a smaller immediate gratification in order to receive a larger one about one week later (Wulfert et al., 2002). Children were presented with incentives and could decide whether they wanted one piece (chocolate chewing gum, tattoo, leapfrog) immediately or larger amounts (two pieces of chocolate, one package of chewing gum, three tattoos, two leap frogs) one week later. We computed the sum of the delayed items (maximum = 4), with higher scores indicating higher self-regulation.

*Affective decision-making*: We used the Hungry Donkey Task (Crone and van der Molen, 2004) to measure children’s tendency to make risky decisions. Children had to collect as many apples as possible for a hungry donkey in 60 trials by choosing one of four doors which revealed a number of lost and won apples. Children chose a door (from left to right) by pressing predetermined letters on a QWERTZ keyboard. Unknown to the children, the doors on the left were disadvantageous in the long run, the doors on the right were advantageous (i.e., providing higher/lower immediate gains, but also higher/lower long-term losses, respectively). We computed a net score of the advantageous minus disadvantageous choices across the last 50 trials, with higher scores indicating higher self-regulation.

*Planning*: Teachers reported on 8 items of the BRIEF (Behavior Rating Inventory of Executive Function, Gioia et al., 2000) plan/organize subscale to measure children’s ability to plan ahead, ranging from (1) *never* to (5) *always*. We computed a mean score after reverse-coding the items, with higher scores indicating higher self-regulation.

### 2.3 Procedure

We approached schools for participation through the headmasters, then single class teachers, and finally children and parents from classes with consenting teachers. We informed all participants about the procedure, including that participation was voluntary and could be terminated at any time without negative consequences. Headmasters and parents provided written consent; children provided assent. We collected a large battery of child experimental data and self-reports in two separate 45-min sessions at school under the guidance of trained research assistants who read out all instructions and explained each experimental task in detail. Parents and teachers answered questionnaires online or via paper-pencil. Since this was a community sample, no exclusion criteria were applied. However, only children in first- to third-grade were eligible for inclusion. Children received several small gifts and a cinema voucher during data collection, parents received information about general study findings, and teachers received €5 per filled-in questionnaire for the class fund. All questionnaires and procedures were approved by the Ethics Committee of the University of Potsdam as well as the Ministry for Education, Youth, and Sports of the Federal State of Brandenburg, Germany. Data collection for T1 took place from 2012 to 2013.

### 2.4 Statistical analyses

In the first step, we conducted descriptive analyses to determine the mean and standard deviation of each self-regulation facet. Next, we tested for differences in the level of self-regulation between the predefined subgroups by performing MANOVAs for each measurement point. We determined partial eta squared (*η^2^*) where *η^2^* ≥ 0.01 indicates a small difference, *η^2^* ≥ 0.06 a medium difference and *η^2^ ≥* 0.14 a large difference (Cohen, 1988). We also conducted Pearson correlations for all included self-regulation measures at T1 and T3. Then, we performed network analyses to gain deeper insights into the patterns of interrelationships between the self-regulation facets. Network analyses illustrate all construct relations in a graphical two-dimensional form. Specifically, each construct is visualized as a circle (“node”), and the relations between the nodes (i.e., the partial correlations excluding the influences of all other nodes) as connecting lines (“edges”). All available self-regulation facets per measurement point were included. The network models were conducted based on recommendations (Epskamp and Fried, 2018; Hevey, 2018) using the R package bootnet (Epskamp et al., 2018, version 1.6), and plotted with the R-package qgraph (Epskamp et al., 2012, version 1.9.8). Networks for subgroups were compared using the R package NetworkComparisonTest (van Borkulo et al., 2023). We estimated an undirected network model using a Gaussian graphical model (GGM; Laureys et al., 2022), which is recommended when the model is built on multivariate normally distributed constructs (Epskamp et al., 2018). To remove spurious edges, we used the LASSO-algorithm (Friedman et al., 2008), with the threshold for the exclusion of spurious edges set as γ = 0.5, following recommendations (Epskamp and Fried, 2018). This approach resulted in a conditionally independent network structure in which the value of a node is partially predicted by all nodes with direct connections (edges), whereas values of nodes without a direct connection do not improve the prediction of this node. To facilitate the graphical comparison between different networks, we fixed the position of each node for all figures based on the complete group network. To examine differences with respect to sex, age, and parental education (each dichotomized as described above), we conducted separate network analyses to determine network invariance and global strength invariance. Each node in a network was described by three centrality measures: *Betweenness* assesses the relevance of a node in connecting other nodes in the network, thus estimating the role a node plays in mediating information between other variables in the network. *Closeness* measures the distance between a node and every other node, thus indicating how a particular node is connected to other nodes. *Strength* measures the number of edges of a particular node and indicates the direct connectivity or “popularity” of a particular node, with higher values indicating higher centrality. We also estimated the global connectivity of the network, i.e., the global strength of each network, with higher values reflecting less differentiation, meaning greater overlap between nodes as well as more and closer interconnections (Epskamp et al., 2018; Hevey, 2018).

## 3 Results

### 3.1 Descriptive statistics and mean differences

Table 2 presents descriptive statistics for all self-regulation facets for the total group and separated by the factors binary sex (female vs. male), age (below vs. above the median), and SES (lower vs. higher parental education) at T1. Supplementary Table 1 shows the descriptive statistics for T3 data. At T1, a MANOVA revealed significant effects of sex, *F*(10, 1038) = 13.51, *p* < 0.001; partial *η^2^* = 0.125; age group, *F*(10, 1038) = 18.73, *p* < 0.001; partial *η^2^* = 0.165; and SES group, *F*(10, 1038) = 9.25, *p* < 0.001; partial *η^2^* = 0.089. Subsequent univariate tests revealed that girls had higher means than boys in inhibition, inhibitory control, flexibility, and planning, as well as lower means in anger reactivity, HRV, and decision-making. Children in the higher age group showed higher means than younger children in inhibition, inhibitory control, working-memory updating, flexibility, delay of gratification, and decision-making. Children in the higher parental education group had higher means than children in the lower parental education group in inhibition, inhibitory control, updating, flexibility, decision-making, and planning, as well as lower means in anger reactivity. Overall, the analyses at T3 revealed fewer differences in mean self-regulation facet levels between the groups, with directions of the differences maintained.

**TABLE 2:** Means, SDs (in brackets), and tests of differences of the main self-regulation facets at T1 by binary sex, age group, and parental education.

Variable	Total	Sex		Age group		Parental Education	
		Girls	Boys	Lower	Higher	Lower	Higher
		M (SD)	M (SD)^†^	M (SD)	M (SD)^†^	M (SD)	M (SD)^†^
EMR	2.21 (0.71)	2.21 (0.69)	2.2 (0.73)	2.18 (0.70)	2.24 (0.72)	2.23 (0.74)	2.19 (0.70)
ANG	2.63 (0.74)	2.56 (0.72)	2.71 (0.75)***	2.63 (0.73)	2.63 (0.75)	2.69 (0.77)	2.6 (0.72)*
INH_B	24.95 (8.78)	25.64 (8.01)	24.2 (9.49)**	22.28 (9.63)	27.64 (6.84)***	24.06 (9.66)	25.53 (8.19)*
INH_Q	3.53 (0.66)	3.62 (0.63)	3.44 (0.68)***	3.52 (0.65)	3.55 (0.68)*	3.39 (0.68)	3.61 (0.65)***
UPD	6.18 (1.47)	6.16 (1.46)	6.2 (1.47)	5.87 (1.43)	6.49 (1.44)***	5.9 (1.42)	6.44 (1.41)***
FLEX	15.58 (4.68)	16.21 (4.68)	14.88 (4.57)***	14.47 (4.67)	16.67 (4.42)***	14.81 (4.82)	16.27 (4.30)***
HRV	58.59 (27.84)	55.73 (26.34)	61.62 (29.05)***	58.35 (26.90)	58.83 (28.76)	59.82 (28.41)	57.96 (27.84)
DOG	0.70 (0.31)	0.68 (0.30)	0.72 (0.31)	0.67 (0.32)	0.73 (0.29)***	0.70 (0.30)	0.71 (0.30)
ADM	5.49 (11.43)	4.01 (9.63)	7.1 (12.93)***	4.73 (11.27)	6.25 (11.55)***	4.15 (10.73)	6.14 (11.73)*
PLAN	3.70 (0.89)	3.89 (0.85)	3.5 (0.90)***	3.69 (0.87)	3.71 (0.92)	3.53 (0.93)	3.91 (0.82)***
*N*	1657	863 (52%)	794 (48%)	830 (50%)	827 (50%)	470 (35%)	864 (65%)

EMR, emotional reactivity; ANG, anger reactivity, INH_B, inhibition; INH_Q, inhibitory control; UPD, updating; FLEX, cognitive flexibility; HRV, heart rate variability; DOG, delay of gratification; ADM, affective decision-making; PLAN, planning.

† The significance of the results from the multivariate analysis of variance (MANOVA) are shown in this column. *p < 0.05, **p < 0.01, ***p < 0.001.

### 3.2 Correlations

Table 3 presents the Pearson correlation matrix of all self-regulation facets at T1. Significant positive correlations emerged between the performance-based measures of inhibition, updating, and flexibility, and parent-reported inhibitory control and teacher-reported planning. There were significant negative correlations between parent-reported anger as well as emotional reactivity and inhibition, inhibitory control, updating, flexibility, and planning. Significant positive correlations were also found between performance-based delay of gratification as well as decision-making, updating and flexibility, and between delay of gratification and inhibition. HRV correlated significantly negatively with inhibition. The correlations at T3 were similar (see Supplementary Table 2).

**TABLE 3:** Zero-order correlations between all self-regulation facets at T1.

Variables	EMR	ANG	INH_B	INH_Q	UPD	FLEX	HRV	DOG	ADM
ANG	0.62***	–	–	–	–	–	–	–	–
INH_B	−0.10*	−0.10*	–	–	–	–	–	–	–
INH_Q	−0.40***	−0.45***	0.11***	–	–	–	–	–	–
UPD	−0.09**	−0.08**	0.27***	0.10***	–	–	–	–	–
FLEX	−0.06*	−0.09***	0.33***	0.14***	0.35***	–	–	–	–
HRV	0.02	0.02	−0.06*	−0.04	0.00	0.00	–	–	–
DOG	−0.05	0.00	0.05*	0.03	0.07**	0.08**	0.05	–	–
ADM	−0.04	−0.01	0.03	0.02	0.07**	0.09***	0.03	0.03	–
PLAN	−0.16***	−0.12***	0.28***	0.30***	0.27***	0.30***	−0.02	0.05	0.01

EMR, emotional reactivity; ANG, anger reactivity; INH_B, inhibition; INH_Q, inhibitory control; UPD, updating; FLEX, cognitive flexibility; HRV, heart rate variability; DOG, delay of gratification; ADM, affective decision-making; PLAN, planning. *p < 0.05, **p < 0.01, ***p < 0.001.

### 3.3 RQ1: network structure

**FIGURE 1:**
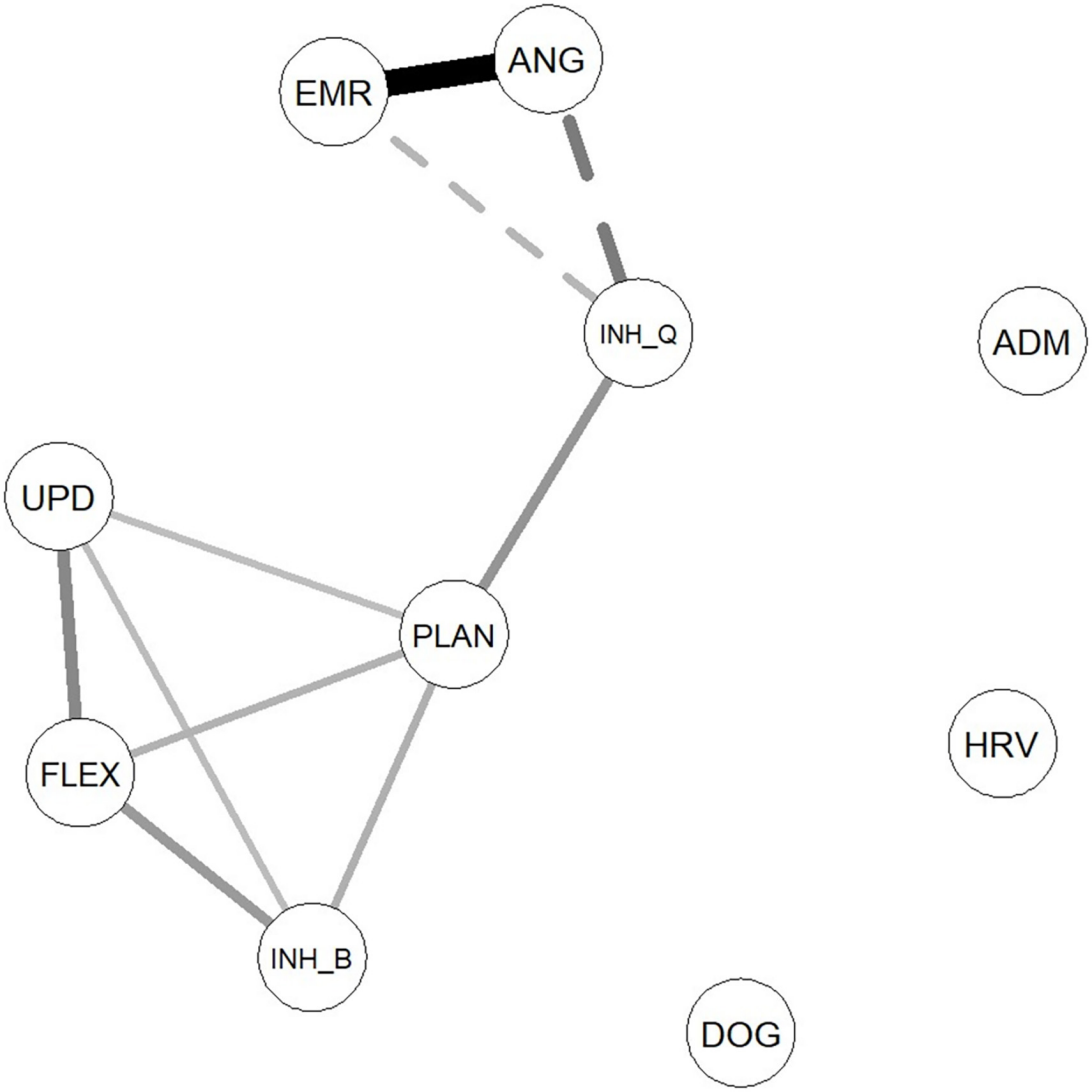
Graphical representation of the network of the self-regulation facets at T1 for the total group. EMR, emotional reactivity; ANG, anger reactivity; INH_B, inhibition, INH_Q, inhibitory control; UPD, updating; FLEX, cognitive flexibility; HRV, heart rate variability; DOG, delay of gratification; ADM, affective decision-making, PLAN, planning. Dashed lines, negative relations; solid lines, positive relations. The thickness of the lines reflects the strength of the respective relation (edge weight).

Figure 1 shows the graphical representation of the network analysis at T1 with 10 nodes (corresponding to the self-regulation facets) and 10 identified relevant edges (links between self-regulation facets). The analysis revealed two connected clusters. The first cluster comprised the basal, affective, parent-reported self-regulation facets emotional reactivity and anger reactivity, with a strong positive edge (weight (*w*) = 0.54)^2^, and basal, cognitive, parent-reported inhibitory control, negatively related to the former two (*w* = 0.15 and *w* = −0.27). Children who were rated by their parents as having better inhibitory control tended to be rated as showing less anger reactivity and general emotional reactivity. The second cluster consisted of basal, cognitive, performance-based inhibition, updating, flexibility, and complex, cognitive, teacher-reported planning, which were all positively connected with edge weights ranging from *w* = 0.13 (updating–planning) to *w* = 0.25 (updating–flexibility). Children ascribed higher planning abilities by their teachers tended to perform better on the behavioral measures of inhibition (*w* = 0.17), updating (*w* = 0.13), and flexibility (*w* = 0.16). An important finding was that the two clusters were connected by a positive edge between basal inhibitory control and complex planning (*w* = 0.22). Children whose parents reported higher inhibitory control tended to be ascribed better planning skills by their teachers. Basal, physiological HRV, complex, performance-based delay of gratification, and decision-making were neither connected to each other nor to any other node. High *betweenness* and *closeness* values indicated that planning was the most relevant node, supporting its role as a bridge between the two clusters (see Supplementary Figure 1). The two basal, parent-reported emotional-reactivity variables reached the highest scores of strength, followed by planning (see Supplementary Figure 2). Regarding network complexity and density, 10 out of the 45 possible edges were considered relevant.

### 3.4 RQ2: moderators

**FIGURE 2:**
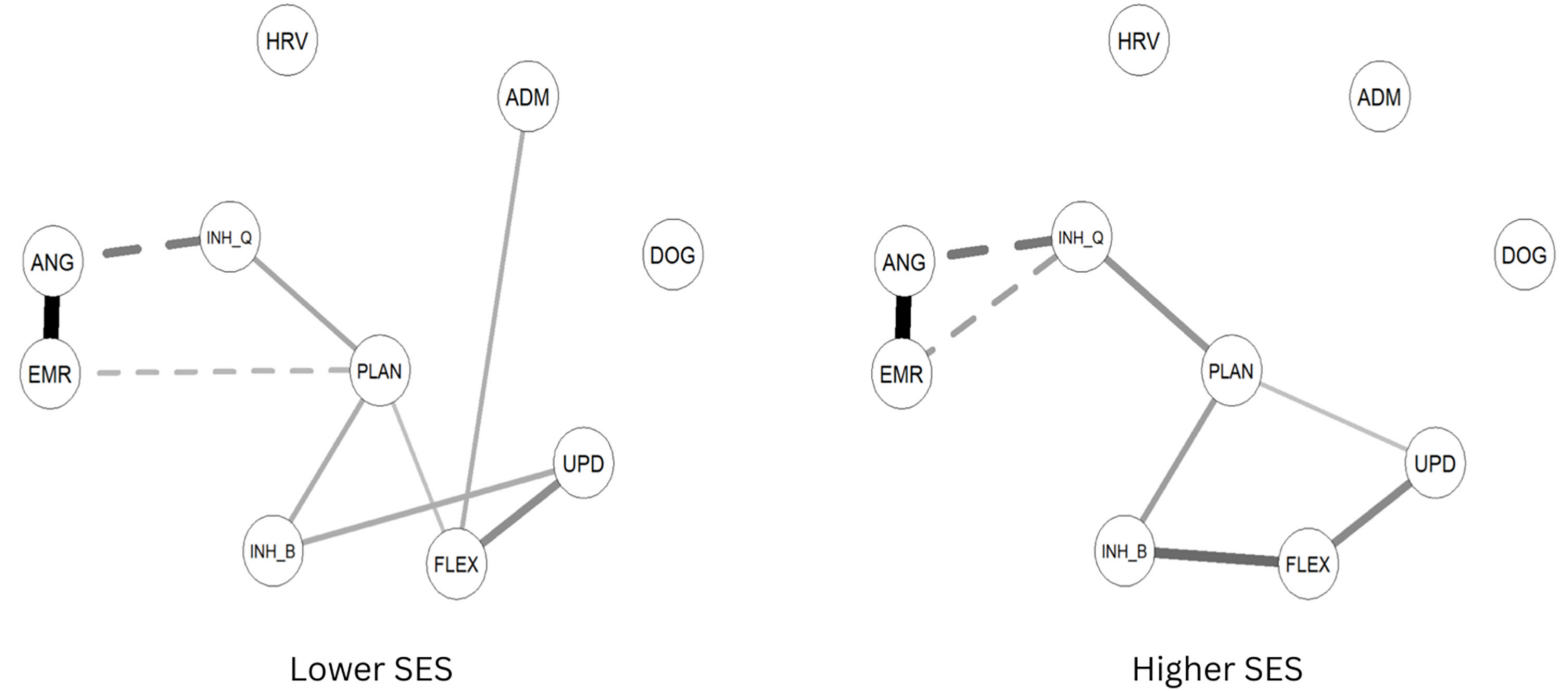
Distinct network analyses of self-regulation facets for the lower and higher parental education group. EMR, emotional reactivity; ANG, anger reactivity; INH_B, inhibition; INH_Q, inhibitory control, UPD, updating, FLEX, cognitive flexibility; HRV, heart rate variability; DOG, delay of gratification; ADM, affective decision-making; PLAN, planning. Dashed lines, negative relations; solid lines, positive relations. The thickness of the lines reflects the strength of the respective relation (edge weight).

Additional analyses at T1 revealed network invariance and global strength invariance for binary sex (network invariance: *M* = 0.17, *p* = 0.43; global strength invariance: *S* = 0.027, *p* = 0.87) and age group (network invariance: *M* = 0.073, *p* = 0.93; global strength invariance: *S* = 0.05, *p* = 0.74), indicating comparable network structures and degrees of differentiation between boys and girls and between older and younger participants at T1. However, the network invariance test indicated a different network structure between the parental education groups (*M* = 0.29, *p* < 0.01), although global strength invariance did not indicate relevant differences in the connectivity of both networks (*S* = 0.03, *p* = 0.35). The graphic representations of the networks for children in the lower and higher parental education-groups are displayed in Figure 2.

By visual comparison, the network structure at T1 was closer to the total group for the higher than the lower parental education group (except for the missing edges inhibition–updating and flexibility–planning). The network analyses revealed a slightly different second cluster (including decision-making) in the lower parental education group: Planning was not only positively connected to inhibitory control but also negatively to emotional reactivity, and emotional reactivity was not linked to inhibitory control. Moreover, there were missing edges (inhibition–flexibility, updating–planning), and an additional edge indicating a particular positive link of the performance-based measures of decision-making (complex) and flexibility (basal) in children with lower parental education. The positive relation between basal, parent-reported emotional reactivity and anger reactivity was stronger in the lower parental education group (w_*lower*_ = 0.61; w_*higher*_ = 0.50, *p* < 0.05).

The same analyses for the T3 data did not reveal any significant differences in the network structures between the sex (network invariance: *M* = 0.17, *p* = 0.52; global strength invariance: *S* = 0.28, *p* = 0.32), age (network invariance: *M* = 0.18, *p* = 0.25; global strength invariance: *S* = 0.35, *p* = 0.19), or parental education (network invariance: *M* = 0.21, *p* = 0.21; global strength invariance: *S* = 0.4, *p* = 0.36) groups.

### 3.5 RQ3: comparisons between T1 and T3

**FIGURE 3:**
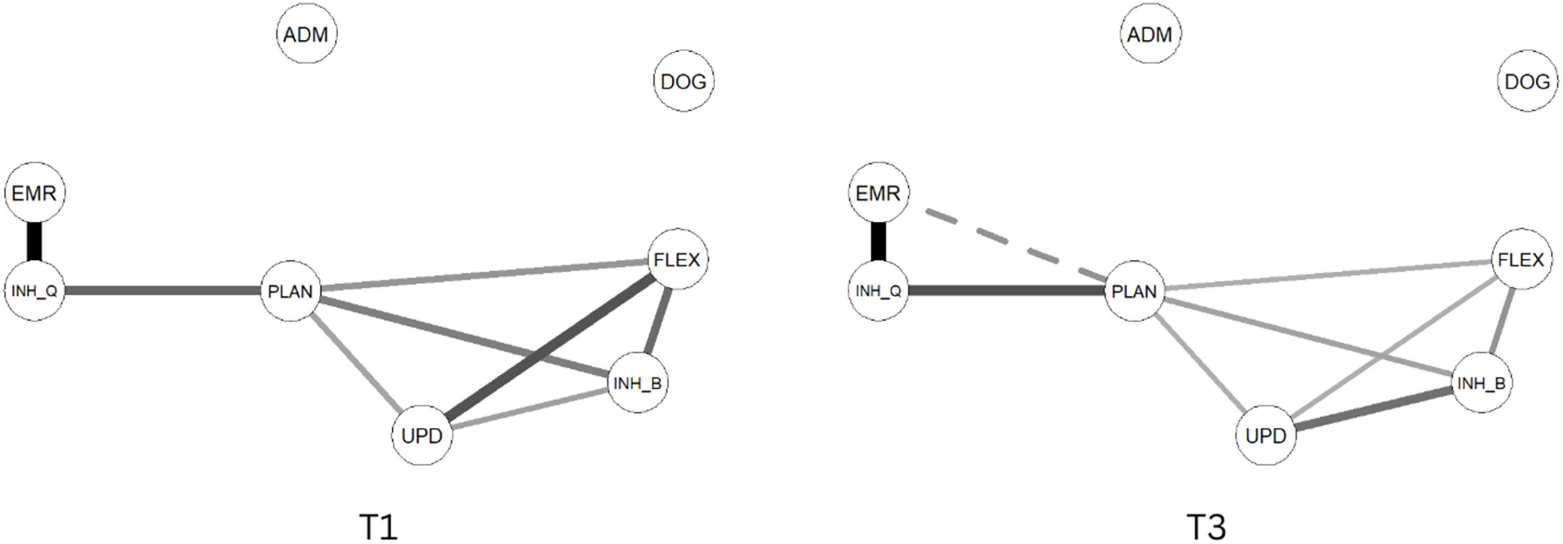
Comparison of the network analyses for the eight included self-regulation facets of T1 and T3. EMR, emotional reactivity; INH_B, inhibition; INH_Q, inhibitory control; UPD, updating; FLEX, cognitive flexibility; DOG, delay of gratification; ADM, affective decision-making, PLAN, planning. Dashed lines, negative relations; solid lines, positive relations. The thickness of the lines reflects the strength of the respective relation (edge weight).

We conducted an additional network analysis for T3 to examine changes in network structure and global node strength over the 3-year period. Correlations among the subfacets and descriptive statistics for T3 are provided in the Supplementary Tables 1, 2). Age-related changes were assessed by comparing the T1 and T3 networks, including the eight self-regulation facets measured at both time points (without HRV and anger reactivity)^3^. The comparison of both networks is displayed in Figure 3.

The overall pattern of the T1 network remained the same as described above. The T3 network was very similar, with one cluster comprising basal, parent-reported emotional reactivity and inhibitory control, and another cluster comprising basal, performance-based inhibition, updating, flexibility, and complex, teacher-reported planning. Again, the complex self-regulation facet planning was the central, connecting node (as indicated by all three centrality indicators, see Supplementary Figure 2), and complex, performance-based delay of gratification and decision-making were – as at the T1 network – unrelated to all other variables. However, the network invariance test yielded the important finding of significant overall differences between the networks at T1 and T3 (*M* = 0.16, *p* < 0.05). First, the T3 network showed an additional negative edge between complex, teacher-reported planning and basal, parent-reported emotional reactivity (*w* = −0.13). Second, the global strength of the nine relevant edges significantly decreased from 1.7 at T1 to 1.3 at T3 (global strength invariance: *S* = 0.34, *p* < 0.05). This was due to decreased strength for three edges (i.e., flexibility—updating, flexibility—inhibition, emotional reactivity—inhibitory control), increased strength for one edge (emotional reactivity—planning), and unchanged strength for five edges. Basal, parent-reported inhibitory control reached the second highest strength and closeness values (see Supplementary Figure 2).

## 4 Discussion

The present study examined the network structure of seven basal and three complex self-regulation facets, assessed across physiological, affective, cognitive, and behavioral foci using parent-/teacher-report questionnaires, physiological or performance-based measures, in a large sample of children (aged 6–11 years at T1) at two measurement points across 3 years. Furthermore, we analyzed potential differences of the self-regulation network structure by binary sex, median-split age, and lower vs. higher parental education (as a proxy for SES). We used network analyses as an analytical tool to understand the complex interrelations between the self-regulation facets, to identify core features, and to create a visual map of the internal structure of self-regulation in middle childhood and adolescence. While several network analyses have been conducted on self-regulation facets in adulthood (Eisenberg et al., 2019; Neubeck et al., 2022), research in childhood is scarce and has focused only on executive functions, neglecting complex self-regulation facets. As an important extension of previous research, we identified a network structure with two clusters of interrelated parent-rated basal affective and cognitive self-regulation facets as well as of performance-based measures of basal cognitive self-regulation facets. The two clusters were connected via teacher-rated planning (a complex, cognitive-behavioral facet) at both T1 and T3. Further self-regulation facets (basal physiological HRV; complex, performance-based cognitive delay of gratification and decision-making) remained unrelated to other facets. The network structure was very similar at T3, but a general decline in the strengths of the edges indicated increasing differentiation between distinct self-regulation competencies across 3 years. Networks of girls and boys as well as of younger and older children were alike, but the structures differed for groups of children with lower vs. higher parental education (as a proxy for SES) at T1 (not T3). This suggests the robustness and reliability of the identified links and the present findings. Due to the centrality of planning, it seems pivotal to address this facet of complex self-regulation in prevention and intervention approaches during childhood and early adolescence.

### 4.1 RQ1: Network structure

We identified two clusters at T1: The first cluster comprised emotional reactivity, anger reactivity (with a strong positive association), and inhibitory control (with negative associations to the first two facets). Notably, all three facets were assessed via parent-report, and they represent affective and cognitive basal aspects of self-regulation in our framework (Table 1; Warschburger et al., 2023). The second cluster consisted of four positively related facets: inhibition, updating, and flexibility, which were measured via performance-based tasks and are seen as core executive functions reflecting basal cognitive aspects of self-regulation (Miyake et al., 2000), and of teacher-reported planning as a complex self-regulation facet encompassing cognitive and behavioral aspects of self-regulation. Planning was also connected to inhibitory control, and was thus the connecting link between the two clusters, which was also evident by its high value of betweenness.

The identification of the first cluster corroborated our a priori considerations: Both general and anger-specific emotional reactivity refer to the child’s tendency to react with intense negative emotions in different situations and can thus be considered as two aspects of the same construct. Their negative association with inhibitory control is consistent with previous research (e.g., Faridi et al., 2015; Geronimi et al., 2016) and underlines associations between the lack of ability to control negative emotions in challenging situations and to restrain counterproductive reactions (Carlson and Wang, 2007; Duijndam et al., 2020; Pruessner et al., 2020). It is important to note that emotional reactivity and anger reactivity had the highest strength values in the network at T1, which can be partly explained by their high direct association (*r* = 0.62; see Table 3). Our findings underscore that the affective component not only plays an important role in the self-regulation network during childhood (age 6–11 years), but also correlates with other facets in the network. This is in line with our framework (Warschburger et al., 2023), postulating that basal affective self-regulation facets provide the basis for the complex, predominantly cognitive-behavioral self-regulation facets (e.g., delay of gratification, decision-making). Other theoretical conceptualizations also assume a hierarchical integrative model of self-regulation, with reciprocal and recursive relations between cognitive, emotional, and behavioral self-regulation competencies (e.g., Blair and Ku, 2022).

The second cluster can be described as a cognitive-behavioral cluster. The observed positive associations between performance-based, basal inhibition, updating, and flexibility are in line with a recent network analysis that also reported positive interrelations between these variables in 7- to 10-year-olds (with comparable edge weights between 0.23 and 0.29; Karr et al., 2022). The close relations are also consistent with the “unity but diversity” concept (Miyake et al., 2000), which posits that these executive functions, or basal self-regulation competencies, can be separated in middle childhood, but are closely related and share common variance. These core executive functions representing mainly basal, cognitive facets of self-regulation were not only highly interrelated but also showed small but positive associations with complex, teacher-reported planning, a relation that has been reported in the literature (Dias et al., 2024; Laureys et al., 2022). Our data brings the complex self-regulation facet planning closer to the three core executive functions. We will discuss the central role of planning in more detail when comparing the network structure at T1 and T3 (see RQ3).

An important finding was the connection of the two clusters via a positive association between the questionnaire measures of planning and inhibitory control, which matches previous findings (e.g., Davidson et al., 2016; Gerst et al., 2017). Consistent with their central role in the network structure, teacher-reported planning and parent-reported inhibitory control had the highest betweenness values of all facets. Notably, these two self-regulation facets are both considered broader phenomena that encompass multiple regulatory foci. However, our undirected network analysis was based on cross-sectional data and thus does not allow for any conclusions on the direction of influence. In summary, our findings extend previous research in important ways, but they also confirm the assumption that complex self-regulation facets are not independent of the expression of basal self-regulation facets, such as executive functions (Bailey and Jones, 2019; McClelland et al., 2018; Warschburger et al., 2023).

The self-regulation facets HRV (basal, physiological; Table 1) as well as delay of gratification and decision-making (both complex, performance-based, affective-cognitive-behavioral) did not engage relevant associations with any of the other self-regulation facets at T1 or T3, aside from the small association of decision-making and flexibility in the lower-SES group at T1, which is consistent with previous findings, based on similar instruments (e.g., Kelly et al., 2020; Miller et al., 2020; Zaccoletti et al., 2023). The reasons for the isolated position of these self-regulation facets in the network need to be further explored. With regard to HRV, meta-analytic evidence (Holzman and Bridgett, 2017) revealed a small, albeit positive association with multiple self-regulation facets (*r* = 0.09) in adults as well as in children and adolescents (*r* = 0.06). However, these analyses did not account for the simultaneous influence of other self-regulation facets, as was done in our network approach (of note, we also found small significant zero-order correlations between inhibition and HRV). Our findings may indicate that our sample was too young to reveal noteworthy associations, which likely emerge only later in development. We conceptualized delay of gratification and decision-making as complex self-regulation facets that require the simultaneous interplay of multiple basal facets (Warschburger et al., 2023; Table 1). Because such an interplay is statistically reflected in nonlinear relations between variables, but network analysis is based on linear relations, the methods of the present study may not have been able to uncover this type of relation.

It is also noteworthy that the two indicators considered to capture the basal ability to suppress impulsive reactions (performance-based inhibition; parent-reported inhibitory control) were not directly, but only indirectly related via complex, teacher-reported planning. The lack of a strong correlation between questionnaires and performance-based measures is well-established in the literature (Duckworth and Kern, 2011) and was also evident in other network analyses (e.g., Eisenberg et al., 2019; Epskamp et al., 2018). Our finding thus provides further evidence that questionnaires and performance-based measures may assess different aspects of self-regulation (e.g., more trait-like or state-like facets; e.g., Doebel, 2020; Ibbotson, 2023).

With regard to core features of the network, the centrality indices revealed a differentiated pattern: Emotional reactivity exhibited the highest strength and thus plays a pivotal role in the network, as discussed above. Planning demonstrated the highest betweenness and closeness scores, thereby emphasizing the function of this complex facet as a bridge between the two clusters, and its robust connections to proximate self-regulation facets. By the inclusion of complex self-regulation facets and the combination of questionnaires and performance-based measures, our study thus extends previous findings that identified either inhibition (Rosales et al., 2023), updating (Holmén et al., 2024), or flexibility (Karr et al., 2022; Menu et al., 2022) as the most central variable. Likewise, highest values of betweenness and closeness were reported for updating (Holmén et al., 2024) or flexibility (Menu et al., 2022). Of note, most of the previous studies concentrated on the core executive functions and used performance-based measures, which underlines the importance of the integrative approach with various self-regulation measures realized in the present study.

### 4.2 RQ2: Moderators

Our study is the first to examine the moderating influence of binary sex, age, and parental education (dichotomized as described above) on the self-regulation networks of the respective subgroups. Although the MANOVA at T1 indicated significant, but only slightly higher levels of self-regulation in girls than boys for most facets (which is in line with the literature; Grissom and Reyes, 2019), our network analyses identified comparable structures of the self-regulation facets at T1 and T3 for both sexes. However, neuroimaging studies (e.g., Gaillard et al., 2021; McKenna et al., 2017) suggest sex-related differences in neural networks that enable self-regulation, which could be matched in further network analyses of questionnaire-based or performance-based self-regulation measures, from childhood to adulthood. Our findings thus call for further network analyses to uncover sex-related differences in the structure of children’s or adolescents’ self-regulation networks.

Our analyses of cross-sectional age effects at T1 and T3 revealed a similar pattern: The MANOVA showed higher self-regulation scores in several facets for the respective older participants (dichotomized by median age: 8.4 years at T1, 11.1 years at T3). This finding is consistent with previous literature (e.g., Fernández-García et al., 2021; Wesarg-Menzel et al., 2023) and indicates an increase in many self-regulation competencies with age (age-related differences were more pronounced at T1). Yet, the network analyses identified comparable structures of the self-regulation facets for the two age groups. The median split enabled a more fine-grained examination of potential age-related changes within a relatively brief age span (approximately 1.5 years). Our results contrast with those of two other studies (Younger et al., 2023; Menu et al., 2024) that observed changes in the organization of executive functions (i.e., basal self-regulation facets) based on visual inspection and centrality measures in children of closer age proximities (cross-sectional 1-year cohorts or longitudinal examinations every 3 months). One study (Menu et al., 2024) also revealed that different tasks, even within the same executive functions, followed distinct developmental trajectories, and another study (Karr et al., 2022) showed changes in global strength, edge weights, and centrality of executive functions in a cross-sectional comparison of age groups aged 3–6, 7–10, 11–13, and 14–17 years. These mixed findings may partly be attributable to the heterogeneity of the included measures and self-regulation facets. In the present study, the detection of age differences may have been impeded by the relatively narrow studied age intervals and by the inclusion of complex self-regulation competencies that should develop later than basal executive functions. Our analyses across T1 and T3 included a broader, 3-year age range and supported this assumption. The present study thus underlines the relevance of longitudinal research to examine potential consistencies in the internal structure of self-regulation facets despite overall developmental improvement.

In contrast to sex and age, our analyses revealed not only significantly higher mean values for several self-regulation facets in participants with higher parental education compared to those with lower parental education, but also significantly different network structures between the two subgroups at T1 (though not at T3). Whereas the network structure for the higher parental education group resembled that of the total group at T1, there were specifics to the lower parental education group, such as a stronger positive connection between the basal facets emotional reactivity and anger reactivity, as well as an additional negative association between basal emotional reactivity and complex planning, and a positive connection between basal flexibility and complex decision-making. While our findings support that parental education is associated with the level of executive functions in youth (Jacobsen et al., 2017; Mousavi and Gharibzadeh, 2021), only one study with 4-year-old-children has examined the influence of parental education on the structure of self-regulation. While they did not find differences in the interrelations between different facets of self-regulation across different levels of parental education, they reported that the mean scores of different basal facets are higher for children of college-educated mothers, even after controlling for their overall level of self-regulation (Montroy et al., 2019). A meta-analysis of neuroimaging studies (Rakesh and Whittle, 2021) found lower activity in neural networks that enable self-regulation and higher activity in the reward network for individuals with lower socioeconomic status (parental education, income or a composite score). Our results suggest a stronger and also direct influence of affective facets within the self-regulation network, although their levels did not differ between the parental education subgroups. In particular, emotional reactivity co-occurs with lower planning competence and a higher tendency to make risky decisions in children of parents with lower education. However, network analyses do not inform about the direction of effects, so further empirical studies are needed to validate this hypothesis. It is important to note that disparities in the self-regulation networks were only identified at T1, not three years later. The influence of parental education on the interaction of self-regulation facets may be particularly pronounced at younger ages, and other factors (e.g., school socialization) may become increasingly prominent over time (Koşkulu-Sancar et al., 2023). The exploration of sensitive periods of self-regulation development is an important avenue for future research, as it will facilitate a more comprehensive understanding of the mechanisms through which parental education influences children’s self-regulation competencies. Specific attention should be paid to parenting as a potential mediating factor (Valcan et al., 2018).

### 4.3 RQ3: comparisons between T1 and T3

We employed a range of analytical strategies to examine the stability or change of self-regulation networks with increasing age. As mentioned above (RQ2), a more nuanced perspective on potential fluctuations by cross-sectional comparisons within the T1 and T3 samples revealed no significant disparities between median-split age groups 1.5 years apart. This may reflect that middle childhood is a period of slower self-regulation growth (Zelazo and Carlson, 2020) and greater stability in network topology. Based on the assumption that differences in relations between self-regulation facets require time to unfold, we also compared networks between T1 and T3, approximately three years apart. Contrary to our fine-grained analysis, but in line with previous studies (e.g., Karr et al., 2022; Menu et al., 2022, 2024; Neubeck et al., 2022), we observed age-related differences in the self-regulation networks at 6–11 and 9–13 years, respectively. In line with the literature and with theoretical considerations (Bardikoff and Sabbagh, 2017; Ibbotson, 2023; Johnson, 2011; Karr et al., 2022), global strength indices suggested a differentiation and specialization of self-regulation facets. A closer look at the edge weights revealed weaker associations between the basal facets of flexibility and both inhibition and updating as well as between inhibition and emotional reactivity, at T3 than at T1. There is only scarce evidence regarding the pattern and timeline of differentiation of self-regulation facets, or even executive functions. However, our data match other network analyses (Hartung et al., 2020; Menu et al., 2022; Rosales et al., 2023), which reported that during childhood, inhibition differentiated first, followed by flexibility. In addition to the decreased strength of the relations at T3, a new edge emerged, showing a negative association between basal emotional reactivity and complex planning. While this association has been found in 7–12-year-old children (Wu et al., 2020; Geronimi et al., 2016), we are not aware of any reported age-related differences in this relation. Despite the innovative nature of our findings, it should be noted that the revealed differences between T1 and T3 were significant, but small. Longitudinal studies spanning wider age ranges and including a variety of self-regulation facets are needed to gain a more detailed understanding of the developmental changes in self-regulation networks.

The central role of planning in the identified self-regulation networks at T1 and T3 aligns with the definition of this complex, cognitive facet as an intentional self-regulation competence that encompasses different (basal or complex) self-regulation facets (Diamond, 2013; Nigg, 2017; Vink et al., 2020). Among others, planning subsumes the abilities to monitor one’s goals (related to updating), to inhibit prepotent reactions for the sake of a superordinate, long-term goal, or to cope with one’s own physiological arousal (emotional reactivity) that may hinder the maintenance of the goal. As a crucial variable between motivational and volitional aspects of behavior, planning is deemed important for health-behavior change (Hagger et al., 2016; Sniehotta, 2009). Our taxonomy considers planning to be a complex facet of self-regulation because it involves strategic thinking to solve a problem or achieve a goal, organizing possible steps in the correct order, and inhibiting automatic impulses. Our finding that the complex facet planning is located at the intersection of the clusters of basal affective and basal cognitive facets underlines the notion that multiple basal competencies are necessary for complex self-regulation (Warschburger et al., 2023). However, as indicated by previous research (e.g., Eisenberg et al., 2019), the present findings may also reflect method- and rater-effects: The first cluster includes only questionnaire-based measures, while the second cluster includes mostly performance-based measures (except for questionnaire-based planning). Of note, planning was rated by teachers who, due to their abundant expertise in assessing children across various domains, are well-qualified to compare children’s planning skills.

### 4.4 Strengths and limitations

To the best of our knowledge, this is the first study that used network analyses to investigate the interrelations of a broad range of self-regulation facets during middle childhood and adolescence. The large community sample of children allowed us to compare the network structure between subgroups with respect to binary sex, age, and parental education, at two measurement points across three years. This study makes an important contribution to the literature on the interrelations of self-regulation facets by including measures of basal and complex self-regulation that cover physiological, affective, cognitive, and behavioral aspects, while providing data for different age groups. With only a few exceptions, we used the same self-regulation facets and established assessment tools across both measurement points.

Besides these major strengths, several limitations should be considered when interpreting the results. First, the network comparisons for RQ3 are based on manifest, cross-sectional data analyses. Second, although we used different methodological approaches (including parent- and teacher-questionnaires, performance-based tasks, physiological measures), we cannot disentangle the role of the self-regulation facet and its method/rater. Therefore, our findings may support the notion that behavioral tasks or questionnaires measure different aspects of self-regulation (i.e., maximum self-regulation capacity for performance-based tasks; usual/trait-like self-regulation in daily life for questionnaires; Doebel, 2020; Saunders et al., 2018). Future studies should adapt a multi-trait multi-method approach to disentangle the psychological construct and its assessment (Duckworth and Kern, 2011; Eisenberg et al., 2019; Holmén et al., 2024). Third, in our analyses we did not account for the nesting of children within schools. While we acknowledge this as a methodological limitation, we believe the negligible effect of school-level clustering, indicated by ICCs mostly below 0.10, suggests that this omission is unlikely to significantly impact the overall results. Since, network analyses are explorative and data-driven replications across comparable as well as differing samples are important in the scientific advance.

## 4.5 Conclusion

The results of the present study provide novel insights into the network of significant self-regulation facets during middle childhood and across three years into early adolescence. The findings suggest that the 10 studied self-regulation facets formed different clusters of basal affective vs. basal cognitive self-regulation facets that may promote complex self-regulation facets such as planning. At the two measurement points, the network structures did not differ between binary sex or median-split age, but parental education had an effect at T1. The identified central role of planning in linking several basal facets of self-regulation suggests that interventions should focus on promoting planning skills, which may also lead to improvements of other self-regulation facets. Further research is warranted to prospectively analyze the dynamics in the self-regulation network across development from childhood to adulthood.

## Data Availability

The raw data supporting the conclusions of this article will be made available by the authors, without undue reservation.

## Footnotes

1 In the PIER-Study, data were also collected at an additional measurement point T2 (about 1 year after T1). Because a change in structure often requires time to unfold, we did not analyze the T2 data. To facilitate the comparison to other papers using this sample, we kept the original labels T1 and T3 for the analyzed measurement points.

2 Because the LASSO approach removed all spurious connections, additional significance testing was not conducted since this could be regarded as double testing

3 Because the edges are based on partial correlations the exclusion of one node affects all remaining nodes. Additionally, we checked whether the age-related variance within a cluster had an impact on the outcomes. Neither for the T1 network (*M* = 0.073, *p* = 0.93), nor for the T3 network age-related moderation could be found (*M* = 0.018, *p* = 0.25).
