# Supplementary material for: Uncovering the structure of self-regulation: a network analysis among German primary-school children
Source: Front Psychol. 2026 Jul 20;17:1823651. doi: 10.3389/fpsyg.2026.1823651 (PMC13430144; doi:10.3389/fpsyg.2026.1823651)
Supplement: Supplementary file 1 [file Table_1.docx]

**Supplementary Material**

**Table S1**
*Means (M), Tests of Differences, and Standard Deviations (SD) of the Main SR Facets at T3 by Binary Sex, Age Group, and Parental Education*

| Variable | Total | Sex | | Age group | | Parental Education | |  |
| --- | --- | --- | --- | --- | --- | --- | --- | --- |
|  |  | Girls | Boys | Lower | Higher | Lower | Higher |  |
|  |  | *M (SD)* | *M (SD)*^†^ | *M (SD)* | *M (SD)*^†^ | *M (SD)* | *M (SD)*^†^ |  |
| EMR | 2.24 (0.73) | 2.28 (0.74) | 2.21 (0.72)*** | 2.22 (0.74) | 2.28 (0.73)*** | 2.27 (0.77) | 2.24 (0.71)*** | |
| INH_B | 35.62 (5.31) | 35.98 (5.27) | 35.22 (5.33)*** | 34.52 (5.53) | 36.73 (4.83)*** | 35.53 (5.31) | 35.73 (5.06)*** | |
| INH_Q | 3.75 (0.68) | 3.82 (0.67) | 3.68 (0.68)*** | 3.75 (0.68) | 3.75 (0.68)*** | 3.59 (0.70) | 3.82 (0.65)*** | |
| UPD | 7.37 (1.63) | 7.36 (1.62) | 7.38 (1.65)*** | 7.23 (1.58) | 7.51 (1.67)*** | 7.17 (1.51) | 7.54 (1.63)*** | |
| FLEX | 9.77 (1.87) | 9.88 (1.91) | 9.64 (1.82)*** | 9.73 (1.84) | 9.80 (1.91)*** | 9.61 (2.03) | 9.87 (1.73)*** | |
| DOG | 0.57 (0.35) | 0.56 (0.34) | 0.58 (0.35)*** | 0.58 (0.34) | 0.56 (0.35)*** | 0.58 (0.33) | 0.58 (0.35)*** | |
| ADM | 9.57 (13.69) | 7.76 (12.33) | 11.53 (14.80)*** | 8.73 (12.70) | 10.40 (14.58)*** | 8.45 (12.87) | 10.07 (14.14)*** | |
| PLAN | 3.67 (0.96) | 3.86 (0.93) | 3.48 (0.94)*** | 3.75 (0.92) | 3.60 (0.98)*** | 3.49 (0.95) | 3.90 (0.89)*** | |

*Note.* EMR = Emotional reactivity, INH_B = Inhibition, INH_Q = Inhibitory control, UPD = Working-memory updating, FLEX = Cognitive flexibility, DOG = Delay of gratification, ADM = Affective decision-making, PLAN = Planning.

^†^ The significances of the results from the multivariate analysis of variance (MANOVA) are shown in this column.

**p* < 0.05. ***p* < 0.01. ****p* < 0.001

**Table S2**

*Zero-order Correlations Between All SR Facets at T3*

| Variables | EMR | INH_B | INH_Q | UPD | FLEX | DOG | ADM |
| --- | --- | --- | --- | --- | --- | --- | --- |
| INH_B | -0.03 |  |  |  |  |  |  |
| INH_Q | -0.34*** | 0.06* |  |  |  |  |  |
| UPD | -0.06* | 0.21*** | 0.07 |  |  |  |  |
| FLEX | -0.01 | 0.17*** | 0.10** | 0.14*** |  |  |  |
| DOG | 0.03 | -0.01 | 0.04 | -0.04 | 0.04 |  |  |
| ADM | <0.001 | 0.03 | 0.02 | 0.08** | 0.03 | 0.03 |  |
| PLAN | -0.21*** | 0.16*** | 0.28*** | 0.16*** | 0.16*** | <0.001 | 0.04 |

*Note.* EMR = Emotional reactivity, INH_B = Inhibition, INH_Q = Inhibitory control, UPD = Working-memory updating, FLEX = Cognitive flexibility, DOG = Delay of gratification, ADM = Affective decision-making, PLAN = Planning.
**p* < 0.05. ***p* < 0.01. ****p* < 0.001

**Figure S1**

*Betweenness, Closeness, and Strength Centrality Measures of the Estimated Network at T1*

*
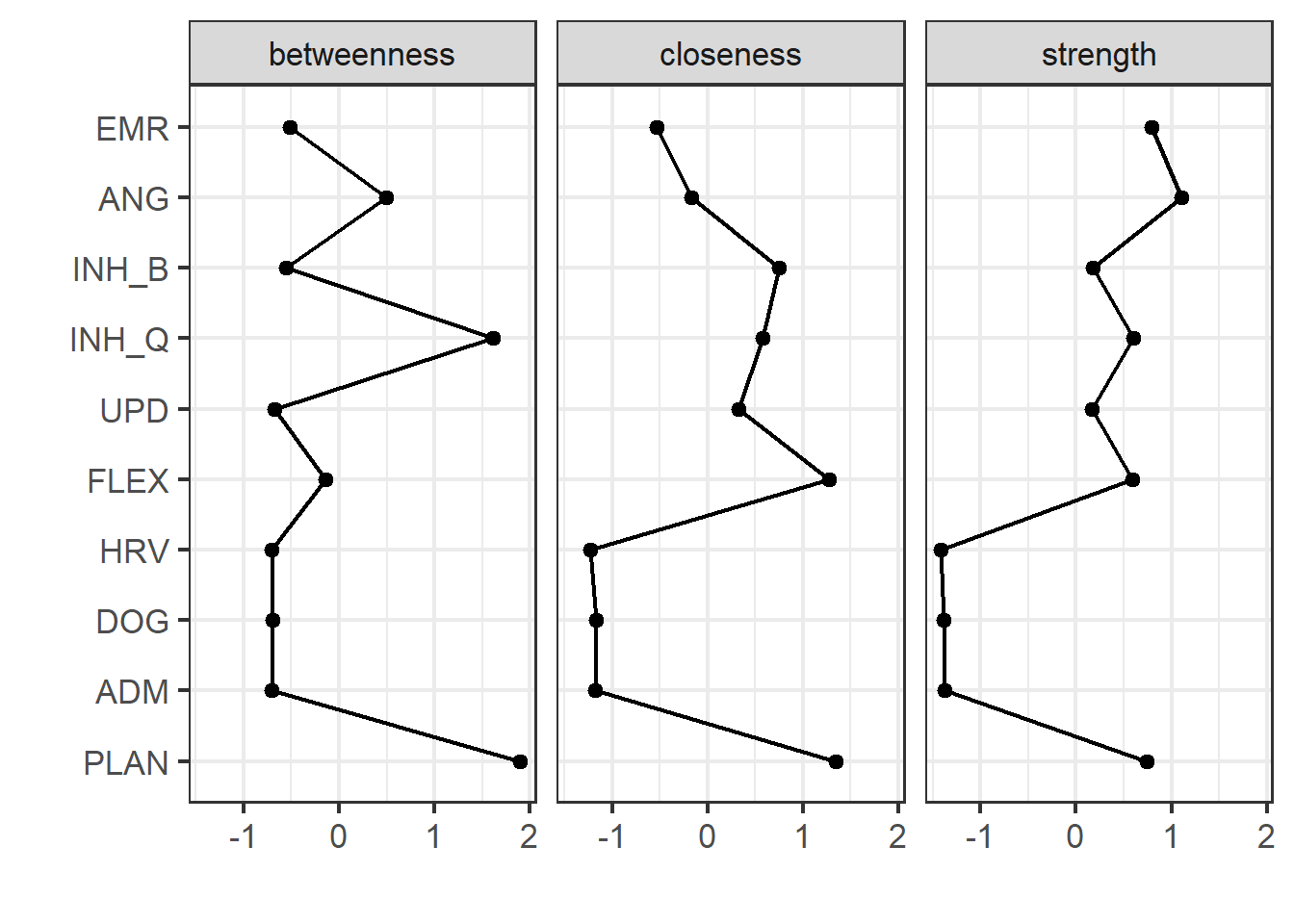
*

*Note.* EMR = Emotional reactivity, ANG = Anger reactivity, INH_B = Inhibition, INH_Q = Inhibitory control, UPD = Updating, FLEX = Cognitive flexibility, HRV = Heart rate variability, DOG = Delay of gratification, ADM = Affective decision-making, PLAN = Planning.

**Figure S2**

*Comparison of the Betweenness, Closeness, and Strength Centrality Measures at T1 (Reduced Network) and T3*


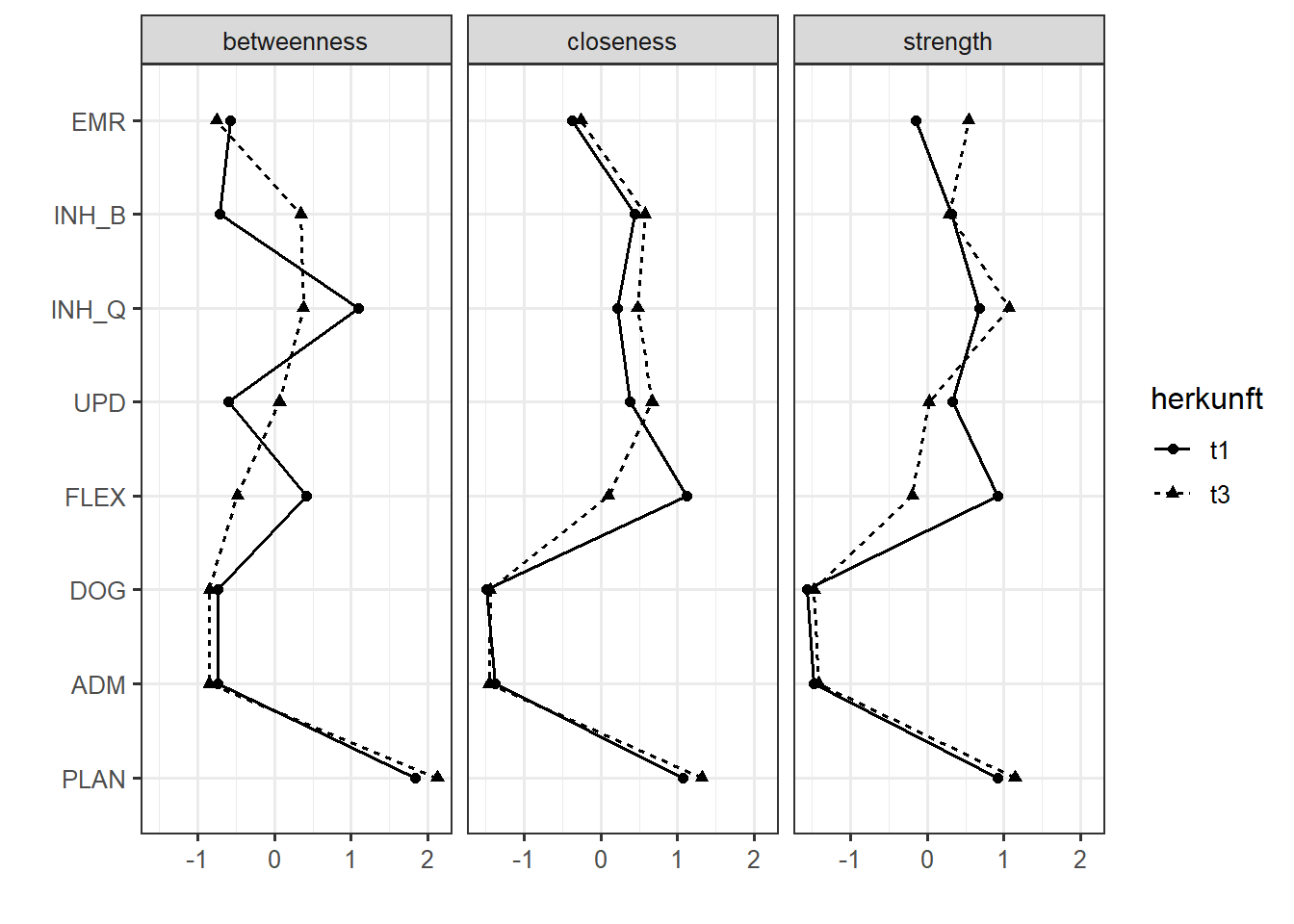


*Note.* Solid lines: centrality measures at T1, dotted lines: at T3. EMR = Emotional reactivity, ANG = Anger reactivity, INH_B = Inhibition, INH_Q = Inhibitory control, UPD = Updating, FLEX = Cognitive flexibility, HRV = Heart rate variability, DOG = Delay of gratification, ADM = Affective decision-making, PLAN = Planning.
